# Editorial: New Perspectives on Pediatric Acute Leukemia

**DOI:** 10.3389/fped.2020.618426

**Published:** 2020-12-07

**Authors:** Riccardo Masetti, Martina Pigazzi, Daniele Zama

**Affiliations:** ^1^Pediatric Hematology and Oncology, Department of Medical and Surgical Sciences, University of Bologna, Bologna, Italy; ^2^Haematology-Oncology Clinic and Laboratory, Department of Woman and Child Health, University of Padua, Padua, Italy; ^3^Department of Medicine, University of Padua, Padua, Italy; ^4^Department of Oncology and Hematology, University Hospital of Bologna Policlinico S. Orsola-Malpighi, Bologna, Italy

**Keywords:** pediatric acute lymphoblastic leukemia, pediatric acute myeloid leukemia, pediatric leukemia, new drugs, acute leukemia biology, targeted therapies

The scenario of pediatric acute leukemia has changed extremely rapidly over these last decade and the pediatric hematologists are now facing new challenges related to the biology of the disease, the prognostic classifications of patients, and the consequent risk-based targeted approach. Acute lymphoblastic leukemia (ALL) in children has always represented a paradigm of success, and the recent advent of large-scale genomic studies and novel immunotherapy-based approaches have further revolutionized the perspective on this disease. Pediatric acute myeloid leukemia (AML) still suffers from a lower cure rate if compared to ALL, due to a still high incidence of recurrence and of severe and dose-limiting short- and long-term toxicities. Nevertheless, much has been learned about the biology of the disease through studies of specific recurrent genetic lesions and the outcomes for these children are progressively improving thanks to the great collaborative efforts of the main pediatric AML groups worldwide.

The Research Topic “New perspectives a in Pediatric Acute Leukemia” includes innovative and original contributions on multiple aspects of pediatric leukemia and gives to the readers the possibility to have an organic and comprehensive overview of novel insights related to the biology of the disease. Original reports aimed at exploring and clarifying the prognostic value of specific recurrent molecular markers are included. A focus on how to refine the patient's risk stratification is covered, with reports on the outcome of specific subgroups of patients such as children with Down Syndrome (DS) or with acute promyelocytic leukemia (APL). Lastly, this collection gives also the opportunity to go deeply into the discovery of novel strategies for targeted therapeutic intervention, from the modeling of pediatric acute leukemia in mice toward a three-dimensional (3D) cell-based drug discovery approach.

The review articles by Kuhlen et al. and Lonetti et al. point out the role of several new drugs targeting key molecular pathways involved in leukemia growth and proliferation that have been developed and approved. These include kinase and proteasome inhibitors, epigenetic and enzyme targeting, as well as apoptosis regulators. Experimental and clinical evidences are comprehensively described and deeply discussed in these contributions, giving a wide and complete overview on the state of the art of novel compounds and targeted therapies currently under evaluation. The contribution by Mercher and Schwaller, provides important insights on new targeted approaches, mainly for AML, but additionally, this review also shed the light on emerging biological concepts of extreme interest. Of particular interest are the data provided on specific subgroups of aggressive AML where the disease phenotype is dependent on the appropriate expression and activity of the driver fusion oncogenes in a particular window of opportunity during fetal development. A novel perspective on the scenario of leukemia drug discovery is also given by the contribution of Cartledge Wolf and Langhans, where interesting dynamic interactions between leukemic cells and the bone marrow environment are explored. The review shows how the interaction between leukemic cells, stromal cells, and the extracellular matrix plays critical roles in the development, progression, and relapse of AML as well as in drug response and the development of resistance.

Moving to the risk based stratification, important aspects related to specific subgroups of patients are also covered. Ksiazek et al. reported an unexpectedly high frequency of the fusion gene transcript resulting from translocation t(10;11)(p12;q23) involving MLL gene, considered an unfavorable prognostic factor, in the Poland pediatric patients affected by AML. As this data was also reported by other reports, it seems that this fusion gene transcript could be relatively frequent in specific populations, opening new considerations on the peculiar biology of this MLL-rearranged leukemia. These characteristics should be taken into account in the analysis of the frequency of recurrent genetic features of pediatric AML.

The paper by Czogala et al. reports the treatment results and genetic characteristics of children with DS affected by AML treated from 2005 to 2019 with two different specific protocols. The study confirms that reduced-intensity protocols are very effective in DS patients with AML without affecting the treatment efficacy. In addition, a significant decrease in treatment-related mortality was noticed, without an increase in relapse. Similar considerations can be made for the other report by the same group (Czogała et al.) reporting the treatment results of a large series of children affected by APL. A good proportion of these children received a treatment regimen that can now be considered the standard of care for standard-risk APL with the combination of all-trans-retinoic acid and arsenic trioxide, without chemotherapy.

In the end, the study of Cwiklinska et al. highlighted the role of pharmacogenetics in the treatment of children with acute lymphoblastic leukemia (ALL). In particular, the authors identified genetic polymorphisms of the *SLC19A1, MTHFR*, and *TS* genes that influence the pharmacokinetics of methotrexate (MTX), increasing the risk of developing hepatotoxicity and vomiting in children with ALL. This study adds an important piece in the comprehension of the entire pathway of the MTX metabolism, that is still far to be fully elucidated. The identification of genetic factors predisposing to the development of specific toxicities could be of great utility in the management of a personalized treatment within the therapeutic protocols.

Overall, this topic highlights that several new perspectives in pediatric acute leukemia are raising and that children suffering from leukemia will have, in a near future, more chances to be cured with innovative approaches and new incoming drugs.

## Author Contributions

RM wrote the manuscript. MP and DZ contributed to the manuscript preparation and critically revised the manuscript. All authors contributed to the article and approved the submitted version.

## Conflict of Interest

The authors declare that the research was conducted in the absence of any commercial or financial relationships that could be construed as a potential conflict of interest.

